# Hypotheses Elicitation in Early-Stage Software Startups Based on Cognitive Mapping

**DOI:** 10.1007/978-3-030-49392-9_14

**Published:** 2020-05-06

**Authors:** Jorge Melegati, Xiaofeng Wang

**Affiliations:** 6 University of Oslo, Oslo, Norway; 7 Monash University, Clayton, VIC Australia; 8 IT University of Copenhagen, Copenhagen, Denmark; 9 University of British Columbia, Vancouver, BC Canada; Free University of Bozen-Bolzano, Bolzano, Italy

**Keywords:** Hypotheses engineering, Software startups, Experimentation

## Abstract

Software startups develop innovative products for which there are typically no customers to refer to elicit requirements. Often, these companies develop a set of features without a better understanding of customer needs. An experiment-based approach to validate hypotheses about the customer and market could increase their chance of success or, at least, accelerate their realization of the product worthlessness. The first step of an experiment-based approach is to elicit hypotheses to guide experiments. Software startups base their products on business assumptions, but there is a lack of understanding of how these assumptions are formed and how teams could elicit hypotheses systematically. To fill this gap, we performed an empirical study consisted of two steps. First, we explored based on which assumptions startups define their products using a multiple case study. The results indicate that these companies developed their products based on founders’ assumptions derived from their previous experience. Second, we investigated cognitive mapping as a tool to elicit hypotheses systematically with two software startups. The results indicate that this approach can serve as the basis of a method to elicit hypotheses in early-stage software startups.

## Introduction

The use of experiments to understand the business value is a recent trend in software engineering [5, 13]. In this context, experimentation is a process of continuously validating product assumptions, transforming them as hypotheses, prioritizing, and testing them following the scientific method to support or refute them [13]. This notion comprises several techniques like prototypes, controlled experiments [5], and problem or solution interviews [13].

In a recent position paper [15], we argued the need for Hypotheses Engineering to handle hypotheses in an experiment-driven approach in a similar way in which Requirements Engineering handles requirements in a traditional software development process. Hypotheses should be elicited, documented, analyzed, and prioritized to perform experiments efficiently. In this paper, we will use “assumption” as a personal or team-wise, generally implicit, understanding taken as truth without being questioned or proved, and “hypothesis” as an explicit statement that has not been verified yet, but an experiment could evaluate. That is, assumptions exist on a cognitive and abstract level, while hypotheses exist on a concrete level in experimentation.

Despite experimentation being a well-known approach for startups and serving as the basis of the Lean Startup methodology [6], software startups still focus on developing the product without testing critical assumptions [9]. In this paper, we targeted the problem of eliciting hypotheses in early-stage software startups, where experimentation is expected to be the primary way of working [17]. The following research question will guide the study: *How can early-stage software startups define hypotheses to support experimentation?*

To achieve our goal, we performed a two-phased empirical study. The first phase aimed to understand how the assumptions on which startups base their products are formed. The second phase investigated how to uncover these assumptions and elicit hypotheses to guide experiments. The first phase results indicated that products are based on the founder’s assumptions about the market and the customer. In the second phase, we used cognitive mapping to make the founders’ assumptions explicit. Our results indicated that this approach could underpin a method to elicit hypotheses systematically in software startups.

## Background and Related Work

Although the term ‘software startup’ is still not a consensus among authors [1], a common set of characteristics has emerged in recent studies: innovation, lack of resources, uncertainty, time-pressure, small team, highly reactive, and rapid evolution [1]. Based on the literature, Klotins et al. [12] proposed a life-cycle model for startups with four stages: inception, stabilization, growth, and maturity. The first stage goes from idea conception until the first release. In the next stage, the startup prepares to scale regarding technical and operational aspects. On these two early-stages, teams focus on finding a relevant problem and solution. In the growth stage, the startup aims to reach the desired market participation, and, in the last stage, it progresses into an established company.

Usually, startups develop software in a market-driven context [1] and offer it to an open marketplace instead of a specific customer. In this latter situation, called specific-customer or bespoke development, one single customer covers the costs to produce the software according to its needs and wishes [16]. Klotins et al. [12] observed the similarities between market-driven development and software startups: mainly invented requirements, light-weight, and informal practices, and quick releases to get customer feedback.

Nevertheless, practices used in the market-driven context may not apply to software startups. In the former, requirements are generally gathered through observing a competing product or collaborating with key customers [16]. In software startups, the options are limited by the innovative nature of products. What makes a product new and unique cannot be found elsewhere. It is typically not recognizable by potential customers, as the phrase attributed to Henry Ford says: “if I had asked people what they wanted, they would have said faster horses.” This mismatch explains why teams in software startups still rely on their ideas or a product team [14] to elicit requirements, especially on how the founder views the market [19]. In such innovative contexts, experimentation has been promoted as an essential practice for new ventures (e.g., [3] and [11]).

In software engineering, experimentation has focused on testing hypotheses about the product and the market [5, 13], and some models were proposed to systematize it [15]. These models extended and are similar to the Lean Startup’s Build-Measure-Learn cycles [17]. In these cycles, startups should first take their assumptions as hypotheses and build the minimum solution to test one of them (Build). Based on metrics (Measure), the team should accept or reject the hypothesis (Learn), that is, persevering or pivoting.

These models provide an overview of the experimentation process, but they do not describe how to define hypotheses [15] and were not explicitly derived for startups. Regarding software startups, to elicit hypotheses, several industry practices have been suggested, such as Business Model Canvas (BMC) (e.g., [8]). Recently, Bland et al. [2] proposed the Assumption Mapping: a set of tools to help teams come up with hypotheses, highly inspired by BMC. But it was not derived from scientific work and did not focus on software startups. In summary, no scientific study focused on how assumptions, on which startups base their products, are formed and how they can inform hypotheses elicitation.

## Research Method

We performed an empirical study divided into two phases, and each consisted of an exploratory multiple-case study. Following the rationale of typical cases [20], we selected software startups in the inception or stabilization phase and where founders had the initial ideas. Through our contact network, we selected four startups (A and B for the first phase, and C and D for the second phase).

The first phase aimed to understand how the assumptions on which startups based their products are formed. It consisted of semi-structured interviews following a defined guide. For both cases, we interviewed the founders and, for case B, also the software developer. The questions aimed to understand the interviewees’ background, the startup idea, motivation to build the product, and how they changed throughout the company history. In the second phase, we evaluated a technique to elicit hypotheses based on the first phase results. It consisted of interviews with startup founders who had the initial ideas. Both founders interviewed in this phase recently did a course where several methodologies and techniques were presented, including Lean Startup and Business Model Canvas.

## First-Phase Results

**Case A.** The startup was developing a software library to be added in projects which will detect run-time problems, like exceptions, observed or inferred based on data collected from the target system. A dashboard will show these problems live along with solutions from similar issues found on the Internet and a list of freelance developers that could help to solve the problem. In some cases, the system would be able to fix some issues automatically. The founder has worked as a software development consultant for an extended period. While working on third-party projects, he observed that such a tool could help him work more effectively. As another reason to develop the tool, he also believed that the technical level of software developers was decreasing nowadays.

**Case B.** The startup runs a website to help hotel owners and managers to find the best software solutions to their businesses. The interviewed founder had worked in a company that handled web marketing and websites before staying twelve years in a big web agency. Throughout his work life, he had extensive contact with the tourism sector, especially the hospitality industry. He claimed that the idea came to him based on the needs he observed from hotel owners, the fact that there are a lot of technological tools available in the market to run the business, and the needs that software vendors have to reach hotel owners. He was inspired by American software review websites and the lack of a specific one for the hospitality sector. Then, the original idea was to list available software with users’ reviews, bring hotel owners to the website, and receive a fee for each lead (an interested customer that visited the vendor website) generated.

When the website went online, the use was below the expected. The team concluded that the hotel owners were not able to compare different solutions because these products rarely have the same set of features, and, often, hotels needed more than one to fulfill their needs. Then, the startup changed the website: now, the hotel owner fills a form giving details about her business, and the system would use a simple algorithm to match solutions with business needs.

**Fig. 1.**
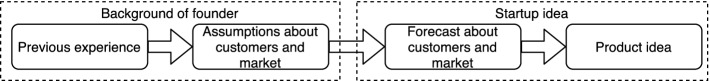
The process of idea creation.

**Cross-case analysis.** Based on the case descriptions above, the founder’s background shaped beliefs about target customers and the market. Through these lenses, founders made sense about the specific business environment and its players, explaining their behavior and, in the last stance, trying to forecast it as illustrated in Fig. 1. Specifically, in startup B, the founder considered that hotel owners wanted to buy software solutions, and they were able to compare different alternatives and select the best for her case. Based on that, the founder foresaw the convenience for hotel owners of a website with the list of available software.

**Fig. 2.**
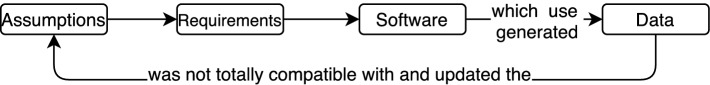
The founder’s assumptions being updated.

The assumptions the founder had about customers and market guided requirements elicitation. In startup B, it was possible to see what could happen next. After the software was ready and put into use, data showed that it was not working as predicted. Therefore, the founder had to update his assumptions and, consequently, change the product. This new understanding emerged from experiments and led to better results. Such rearrangement exposed an implicit process model (see Fig. 2) for development in software startups: the founder’s assumptions guide the elicitation of requirements and the software usage data may impose changes on these assumptions. This updated world representation is used to elicit new requirements.

**Cognitive mapping.** To further explore the founders’ assumptions, a valuable approach would be to make them explicit. For this task, an available tool is cognitive mapping. Cognitive maps are visual representations of causal aspects of a person’s belief system as a graph where nodes represent the concepts individuals use and arrows, causal links between them [7] labeled according to its association: ‘+’, positive; ‘−’, negative, and ‘/o/’ neutral.

**Fig. 3.**
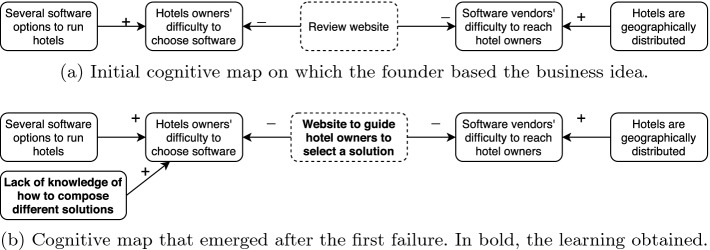
Startup B founder’s assumptions in different moments of the company life.

We used case B to illustrate the approach. First, we elicited the founder’s initial cognitive map (Fig. 3a). Then, through the relationships among concepts in the map, we derived hypotheses on which the product was based. They are (1) owners have several software options to run hotels; (2) because of that, they have difficulty to choose software; (3) a list of options would help owners to select the product; (4) software vendors have difficulty to reach hotel owners. Hence, the first product version acted as an experiment to test the usefulness of a list of available software to hotel owners, which results made the founders update their assumptions about the customers’ behavior (Fig. 3b). Such analysis was performed *ex post* (after the product was developed). To verify if a cognitive map could be used *ex ante*, we performed a second phase for this study.

## Second-Phase Results

In this phase, we performed a study with two other software startups, C and D. We interviewed the founders following the steps: (1) present the hypothesis concept and its relation to Lean Startup; (2) ask a summary of the startup idea, focusing on customer segments and value proposition; (3) ask on which hypotheses the founder believed his idea is based; (4) using a whiteboard and interacting with the founder, draw a cognitive map; (5) create a list of hypotheses based on the cognitive map and compare it with the initially created list; (6) ask feedback.

**Fig. 4.**
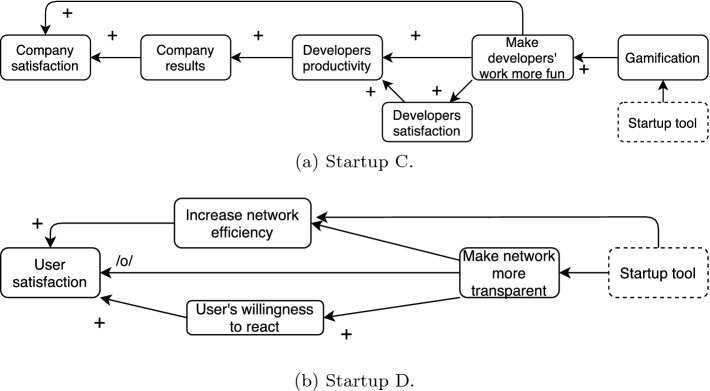
Cognitive maps created during interview with the founders.

To draw the map, we adapted the approach proposed by Furnari [7]. First, we asked the interviewee to describe the business model. From that, we extracted concepts and causal relationships. Then, we dig on each concept to see if they were, in reality, not based on an underlying assumption. The process ended when the interviewee said that the map represented her understanding of the problem. Throughout the process, we used the whiteboard to depict the current status of the mapping. Figure 4a and b display the cognitive maps obtained.

**Case C** is a startup where the founders plan to develop a digital mentor for software developers to increase their happiness and satisfaction. The product would try to adapt itself to each developer’s needs. The paying customer would be companies interested in improving their developers’ productiveness. When asked about hypotheses, the founder mentioned that the first was that software development teams could not organize themselves. Through some customer interviews, it got invalidated, and they pivoted the initial idea to the current one. The next hypothesis or, how the founder called, “exploration” is to understand if software developers care about soft skills. When asked about other hypotheses, the founder said that she is waiting for another round of tests.

In the interview, the founder stated that the main element to increase developers’ productivity would be making their work more fun through gamification. The map implied six hypotheses: (1) developers productivity improves the company results; (2) developers satisfaction rises developers productivity; (3) making the development work more fun increases the developers’ productivity and (4) the developers’ satisfaction; (5) gamification could make developers’ work more fun; (6) making the development work more fun would rise the company satisfaction. Although some identified hypotheses are trivial and may not demand an experiment, the founder recognized that “[they] have to see if the correlation between having fun and the productivity [exists], that is a major risk.”

**Case D** is developing a solution to improve network connectivity, especially where the Internet quality is low. Through an innovative approach, suppressed here according to the interviewee’s request, the solution will make the network status transparent to the user, allowing it to be adapted to the needs and, consequently, improving the quality of service. Initially, the founder answered that their main hypothesis regarded how large is the area where the quality is bad and if providers are willing to fix it soon. He mentioned that he talked to many potential customers, and most of them would want the solution.

The map implied four hypotheses: (1) increasing the network efficiency will improve user satisfaction, (2) making the network more transparent will not decrease user satisfaction, (3) making the network more transparent will increase the user’s willingness to react, and (4) the users’ willingness and ability to respond will increase user satisfaction. The founder mentioned they had considered these hypotheses before, but the process “made them explicit and more structured.”

## Discussion

Software startups elicit requirements on their own, based on assumptions regarding the customer or market. Since the founder is generally the sole owner of innovation [19], these assumptions are based on founders’, not necessarily explicit beliefs. In other words, products are based on the founders’ tacit knowledge about the customer and market. Yet, the delay in abandoning an unworthy idea could mean exhausting the resources and, consequently, failing the company. The possible reasons not to make these assumptions explicit include protecting them against criticism [4] or avoiding an uncomfortable situation of not being able to predict and control if they are invalidated [10]. Besides that, founders try to predict a distant event: the use of a product or a service after development. Thus, as we observed, it can take time for the founder to review her assumptions. Such an adjustment is more frequent when used to predict immediate happenings [10].

A hypothesis elicitation method should evidence assumptions that are guiding the startup product development. Then, the first step is to make explicit the founders’ assumptions. Our study showed that cognitive mapping is a viable way to do it. This option is related to what Furnari [7] called “cognitive perspective” in business model research.

The value of a model cognitive map is two-folded. First, it allows the startup to check the business model for flaws. Second, the team can derive hypotheses from the cognitive map for experiment creation. Data collected from experiments will validate their understanding or update it. Once ideas are validated, they can be used to guide requirements elicitation.

To handle the threats to validity, we followed the definitions given by Runeson et al. [18]. Since the interview guide focused on the business model description and evolution, the threat to construct validity is minimal. Besides that, the triangulation of data with interviewing a different team member decreased the threat even more. Triangulation was also essential to mitigate threats to internal validity. Besides that, both authors discussed the results (peer debriefing). Concerning external validity, in case studies, it is not possible to draw statistical significance [18]. Then, the goal was analytical generalization through studying typical software startups where the founder is the main innovation owner. These companies generally focus on developing a solution instead of understanding the customer [9]. To improve reliability, we described all the performed steps.

## Conclusions

Early-stage software startups have to evaluate if their ideas are worth pursuing. Developing experiments based on hypotheses about various aspects of a business model is essential to this task. In such a process, the first step is to define the hypotheses. There are some techniques in the literature to perform this, but they were not systematically obtained from scientific knowledge. To derive a basis for such a tool, we conducted a two-phased empirical study. First, we concluded that the founder’s past experiences mold a set of assumptions used to predict the environment and how a new product would behave. Then, we had promising results using cognitive mapping to elicit hypotheses leading us to believe it could serve as the basis of a method for early-stage software startups. In order to develop such a method, future work should answer some questions, such as if the tool can elicit all hypotheses related to the product.
